# The influence of calcium-phosphate metabolism abnormalities on the quality of life in patients with hemodynamically significant mitral regurgitation

**DOI:** 10.1186/s12872-019-1094-3

**Published:** 2019-05-16

**Authors:** Olga Mozenska, Jacek Bil, Agnieszka Segiet, Dariusz A. Kosior

**Affiliations:** 1 Department of Cardiology and Hypertension, Central Clinical Hospital of the Ministry of the Interior and Administration, Woloska Street 137, 02-507 Warsaw, Poland; 2 Department of Internal Medicine, Hypertension and Vascular Diseases, Warsaw Medical University, Banacha Street 1a, 02-097 Warsaw, Poland; 3 Department of Invasive Cardiology, Centre of Postgraduate Medical Education, Central Clinical Hospital of the Ministry of the Interior and Administration, Woloska Street 137, 02-507 Warsaw, Poland; 4 Department of Applied Physiology, Mossakowski Medical Research Centre, Polish Academy of Sciences, Pawinskiego Street 5, 02-106 Warsaw, Poland

**Keywords:** Vitamin D, Hypocalcemia, Valvular heart disease, MacNew, parathormone, Quality of life, Health-related quality of life

## Abstract

**Background:**

In recent years, studies have indicated that vitamin D [25(OH)D_3_] and other calcium-phosphate (Ca-P) metabolism parameters and their disturbances might be potential new factors that may influence health-related quality of life (HRQoL). The aim of our study was to assess the extent of Ca-P metabolism abnormalities in patients with significant mitral regurgitation (MR) and their effect on patients’ HRQoL.

**Methods:**

We included 99 patients with significant MR (median age, 75 years [Q1–Q3, 66.0–81.5], 35.4% females). Hemodynamically significant MR was assessed using transthoracic echocardiography (vena contracta > 3 mm, effective orifice area > 0.2 cm^2^, and MR volume > 30 mL/s). HRQoL was evaluated using a cardiac-specific (MacNew) tool.

**Results:**

A significant negative correlation between parathormone (PTH) levels and HRQoL was demonstrated (r = − 0.242, − 0.243, and − 0.255; *p* = 0.018, 0.018, and 0.013 for Global Scores, and physical and social domains, respectively). Additionally, we confirmed that patients with higher NT-proBNP levels, NYHA heart failure (HF) class, and larger left ventricles had poorer HRQoL. Moreover, patients with poorer HRQoL walked a shorter distance in a 6-min walking test.

**Conclusions:**

To the best of our knowledge, this report is the first to show that Ca-P abnormalities resulted in significantly worse HRQoL, especially in the physical domain, in a population of patients with hemodynamically significant MR.

## Background

Valvular heart disease, after coronary artery disease and hypertension, is the third most common cause of heart failure (HF). In Europe, mitral regurgitation (MR) is the second most frequently acquired valve disease in adults [1]. As the population ages and life expectancy increases, living a healthy and longer quality life is becoming more important for communities and individuals [2–4]. Along with that comes a wish to address ways for improving health-related quality of life (HRQoL). In patients with MR, several factors have been associated with HRQoL, such as impaired left ventricular function or end-systolic diameter of the left ventricle ≥45 mm [5–7]. One should also consider HRQoL depending on the repair method [8, 9]. Moreover, the psychoemotional status has proven to influence HRQoL [10–12]. In recent years, studies have indicated that vitamin D [25(OH)D_3_] and other calcium-phosphate (Ca-P) metabolism parameters and their disturbances might be potential new factors that may influence HRQoL. Considering the frequencies of Ca-P metabolism abnormalities and MR in general and in the cardiovascular population, we aimed to assess the influence of Ca-P metabolism disturbances on HRQoL in this type of population.

## Methods

### Study population

The study population consisted of 99 patients hospitalized in our Cardiology Department between July 1, 2013 and September 30, 2013 [13]. The patients were hospitalized on an elective basis and in an emergency. Summer months were typically chosen to ensure feasible and constant exposure to sunlight. The study inclusion criteria were age over 18 years and significant MR assessed using transthoracic echocardiography (vena contracta > 3 mm, effective orifice area > 0.2 cm^2^, and MR volume > 30 mL/s). The exclusion criteria were lack of written informed consent, prior medical history of invasive MV treatment (surgical or percutaneous MV repair and surgical MV replacement), MV stenosis of any kind, chronic kidney disease requiring dialysis, prior medical history of thyroid or parathyroid gland disorders (including status post thyroidectomy or post parathyroidectomy), clinical situation requiring temporary (at least 2 weeks) or permanent immobilization 6 months before being included in our study, and routine use of Ca or vitamin D supplements. At the time of hospital admission, we performed physical examination, obtained anthropometric measurements, and collected detailed prior medical history. We concentrated on valvular heart disease, Ca-P metabolism, vitamin D deficiency, dietary regimens, physical activity, and vitamin supplements. The course of each hospitalization was standard and adequate to determine symptoms, clinical status, initial diagnosis, and comorbidities.

### Biochemical parameters

Blood specimens were obtained in a standardized fashion in the morning, after at least 12 h of fasting and 30 min of resting in a supine position in a quiet, environmentally controlled room. Fasting morning urinary samples and 24-h urinary samples were collected. The serum PTH levels were determined using Elecsys PTH (1–84) test (Cobas®, Roche Diagnostics GmbH, Germany). Vitamin D (25(OH)D_3_) levels were measured using the LIAISON test (DiaSorin Inc., USA). To calculate [Ca]_alb_ and [Ca^2+^]_pH_, we used the standard formulas [14, 15].

The laboratory calibration references for Ca-P metabolism parameters were as follows: [Ca]_alb._ (serum), 2.19–2.54 mmol/L; [Ca^2+^]_pH_ (serum), 1.13–1.32 mmol/L; daily urinary Ca secretion, 2.5–6.25 mmol/24 h (females) and 3.75–7.5 mmol/24 h (males); phosphates (serum), 0.9–1.5 mmol/L; phosphates (urine), 40–136 mg/dL; daily urinary phosphate secretion, 0.4–1.3 g/24 h; PTH (serum), 10–65 pg/mL; and 25(OH)D_3_, 30–80 ng/mL.

### Echocardiographic parameters

A comprehensive, standard, two-dimensional Doppler echocardiography examination was performed using a commercially available diagnostic ultrasound system (iE 33, Philips Medical System, Best, The Netherlands). All the measurements were obtained by an experienced cardiologist, in accordance with the guidelines of the European Association of Cardiovascular Imaging [16, 17].

### Quality of life assessment

HRQoL was assessed using a disease-specific instrument (MacNew) [18–20]. The MacNew questionnaire is a self-administered HRQoL assessment tool developed for patients with myocardial infarction, HF, and angina pectoris. It consists of 27 items evaluated by patients using a 7-point Likert scale (little or no symptoms, 7; severe symptoms, 1). A Global Score is calculated as the average score of three domains (social, emotional, and physical). Permission to use was obtained.

### Research ethics and patient consent

The study was conducted in accordance with the World Medical Declaration of Helsinki. All study subjects provided their written, informed consent for participating in the study. The Institutional Review Board approved the study protocol (approval number 63/2013, issued July 1, 2013).

### Statistical analysis

The Shapiro–Wilk test of normality was used to assess the distribution of continuous variables, and continuous variables were reported as the mean ± standard deviation values for normally distributed variables. Median [first quartile–third quartile] was reported for the variables deviating from normal distribution. Categorical variables were reported as frequencies and percentages. To compare continuous variables, Student’s *t*-test was used for normally distributed variables, and Mann–Whitney test was used for variables deviating from normal distribution. Categorical variables were compared using Fisher’s exact test. The influence of selected clinical, echocardiographic, and biochemical variables on HRQoL was assessed using univariable and multivariable logistic regression models. The significance level was set at 0.05. Statistical analysis was performed using R 3.1.2 statistical package (R Core Team (2014). R: A language and environment for statistical computing. R Foundation for Statistical Computing, Vienna, Austria. Available at http://www.R-project.org/).

## Results

### Baseline characteristics

We screened 376 potentially eligible patients, of whom 257 did not meet the study criteria, and 20 patients did not sign the informed consent form. Ultimately, we enrolled 99 patients with a median age of 75 years (IQR, 66.0–81.5). All study subjects had HF (NYHA I, 36.4%; NYHA II, 34.3%; NYHA III, 11.1%; and NYHA IV, 18.2%); 64.6% had hypertension, and 46.5% had chronic kidney disease. The clinical characteristics of study patients and detailed echocardiographic and biochemical parameters are presented in Tables 1 and 2. The median serum level of calcium corrected by albumin was 3.22 mmol/L (IQR, 3.14–3.27), and the mean serum phosphate level was 3.51 ± 0.62 mmol/L. The median serum PTH level, which was 63.10 pg/mL (IQR, 40.95*–*88.55), was abnormally elevated in 48.42% of the study population. In 92.71% of the study cohort, we acknowledged 25(OH)D_3_ deficiency. Detailed Ca-P metabolism components and frequency of their disturbances are shown in Table 3.

**Table 1:** Study population characteristics

Parameter	Study group n (%) / mean ± SD / median [IQR]
Total number of patients:	*N* = 99
Age (years)	75.0 [66.0–81.5]
Female	35 (35.4)
Weight (kg)	80.0 [67.0–87.8]
Body mass index (kg/m^2^)	26.5 [24.2–30.2]
Body surface area (m^2^)	1.89 ± 0.25
Comorbidities:	
HF with preserved ejection fraction	32 (32.3)
HF with reduced ejection fraction	44 (44.4)
Right ventricular HF	25 (25.3)
Dilated cardiomyopathy	36 (36.4)
Arterial hypertension	64 (64.6)
Diabetes	32 (32.3)
Chronic kidney disease	46 (46.5)
stage I – II	60 (60.6)
stage III - IV	39 (39.4)
Atrial fibrillation	39 (63.9)
Osteoarthritis	24 (24.2)
Distance in 6-min walking test (m):	150.0 [40.0–440.0]
Pharmacotherapy:	
Beta-blockers	85 (87.6)
ACEi	78 (80.4)
ARBs	11 (11.3)
Ca channel antagonists	20 (20.6)
Diuretics	76 (78.4)
Mineralocorticoid receptor antagonists	35 (36.1)

*ACEi, angiotensin converting enzyme inhibitors; ARBs, angiotensin receptor blockers; HF – heart failure*

**Table 2:** Baseline echocardiographic and laboratory findings

Parameter	Study group mean ± SD / median [IQR]
Echocardiography:	
LVEF (%)	50.0 [29.0–62.0]
LVEDd (mm)	56.0 [49.5–65.0]
LVED_vol_ (mL)	111.5 [79.0–182.3]
LVED_vol_ /BSA	58.4 [43.2–91.4]
IVSDd (mm)	10.0 [10.0–11.8]
PWDd (mm)	10.0 [9.0–11.0]
LA_vol._ (mL)	100.0 [71.3–126.0]
LA_vol._/BSA	53.0 [38.2–65.5]
RVSP (mmHg)	43.0 [39.0–53.5]
TAPSE (mm)	22.0 ± 6.8
Mitral valve annulus (mm)	37.4 ± 6.4
Vena contracta (mm)	6.0 [5.0–7.0]
PISA radius (mm)	6.0 [5.0–7.0]
Mitral regurgitation volume (ml/beat)	23.0 [17.0–31.0]
ERO (cm^2^)	0.15 [0.10–0.22]
Laboratory tests:	
creatinine (mmol/L)	1.03 [0.83–1.22]
eGFR (mL/min/1.73 m^2^)	67.11 ± 24.09
albumins (g/L)	4.00 [3.72–4.22]
total proteins (g/L)	6.52 ± 0.69
C-reactive protein:(mg/L)	2.80 [1.10–8.47]
NT-proBNP (pg/ml)	1815.0 [583.5–3483.3]

*BSA, body surface area; LVEF, left ventricular ejection fraction; LVDd, left ventricular diastolic diameter; LVED*_*vol*_ *, left ventricular end-diastolic volume; IVSDd, intraventricular septum diastolic diameter; PWDd, posterior wall diastolic diameter; LA*_*vol.*_ *, left atrial volume; RVSP, right ventricular systolic pressure; TAPSE, tricuspid annular plane systolic excursion; PISA, proximal isovolumetric surface area; ERO, effective regurgitant orifice area*

**Table 3:** Calcium-phosphate metabolism components and their incidence frequency in the studied population

Parameter	Study group mean ± SD / median [IQR]	Frequency of deficiency [%]	Frequency of excess [%]
[Ca]_alb._ (mmol/L) (serum)	3.22 [3.14–3.27]	0	100
[Ca^2+^]_pH_ (mmol/L) (serum)	1.05 ± 0.08	84.3	0
Calcium 24-h urinary excretion (mmoL/24 h)	2.25 [1.28–2.92]	56.6	4.0
Phosphates (serum) (mmol/L)	3.51 ± 0.62	0	100
Phosphates (urine) (mg/dL)	30.00 [18.15–37.65]	76.0	2.7
Phosphates 24-h urinary excretion (g/24 h)	0.47 [0.32–0.75]	36.0	2.7
PTH (serum) (pg/mL)	63.10 [40.95–88.55]	0	48.4
25(OH)D_3_ (ng/mL)	14.80 [9.93–20.12]	92.7	0

*Ca, calcium; PTH, parathormone*

### Influence of ca-P parameters abnormalities on HRQoL of patients with hemodynamically significant MR

We observed a significant negative influence of the increase in PTH levels on the Global Score (r = − 0.242; *p* = 0.018) and HRQoL in physical and social domains (r_1_ = − 0.243, p_1_ = 0.018; r_2_ = − 0.255; p_2_ = 0.013 for physical and social domains, respectively). To determine the exact influence of Ca-P metabolism parameters on individual MacNew domains, we compared HRQoL scores reported by patients in two subgroups with and without disturbed Ca-P metabolism parameters. In the subgroup with elevated PTH levels, the mean Global Score was significantly lower than that in the subgroup with PTH levels within the normal range (4.05 ± 1.29, 4.34 ± 1.26, and 3.76 ± 1.39 for the entire study population, subgroup with normal PTH levels and subgroup with elevated PTH levels, respectively; *p* = 0.031). Also, the median scores in the physical and social domains were significantly lower in the subgroup with elevated PTH levels (physical domain: 3.58 [2.65–4.83], 3.69 [2.69–4.83], and 3.15 [2.73–4.96] for the entire study population, subgroup with normal and subgroup with elevated PTH levels, respectively; *p* = 0.027; social domain: 3.92 [3.00–5.23], 4.46 [3.31–5.54], and 3.50 [2.79–4.73] for the entire study population, subgroup with normal and subgroup with elevated PTH levels, respectively; *p* = 0.019). Regarding other evaluated Ca-P metabolism parameters, no significant influence on HRQoL was observed. The results of Ca-P metabolism parameters and their influence on HRQoL are presented in Tables 4 and 5.

**Table 4:** Calcium-phosphates metabolism parameters and their influence on health-related quality of life in patients with significant mitral regurgitation

Calcium-phosphate metabolism parameter	MacNew domain	Spearman’s rank correlation coefficient	*p*-value
[Ca]_cor.albumins_ (mmol/L) (serum)	Global Score	−0.043	NS
	physical	−0.019	NS
	emotional	−0.089	NS
	social	−0.017	NS
[Ca^2+^]_cor.pH_ (mmol/L) (serum)	Global Score	0.062	NS
	physical	0.081	NS
	emotional	0.044	NS
	social	0.063	NS
Calcium 24-h urinary excretion (mmoL/24 h)	Global Score	−0.030	NS
	physical	−0.056	NS
	emotional	−0.003	NS
	social	−0.012	NS
Phosphates (urine) (mg/dL)	Global Score	0.051	NS
	physical	0.074	NS
	emotional	0.038	NS
	social	−0.006	NS
Phosphates 24-h urinary excretion (g/24 h)	Global Score	0.097	NS
	physical	0.128	NS
	emotional	0.098	NS
	social	0.063	NS
PTH (serum) (pg/mL)	Global Score	−0.242	0.018
	physical	−0.243	0.018
	emotional	−0.176	NS
	social	−0.255	0.013
25(OH)D_3_ (ng/mL)	Global Score	0.157	NS
	physical	0.189	0.066
	emotional	0.122	NS
	social	0.161	NS

*Ca, calcium; PTH, parathormone*

**Table 5:** Calcium-phosphates metabolism parameters and their disturbances and their influence on health-related quality of life in patients with significant mitral regurgitation

Calcium-phosphates metabolism parameter	MacNew domain	Study population mean ± SD / median [Q1-Q3]	No disturbance mean ± SD / median [Q1-Q3]	Disturbance present mean ± SD / median [Q1-Q3]	*p*-value
[Ca]_kor.albumins_:(mmol/L) (serum)	Global Score	4.05 ± 1.29	4.08 ± 1.41	4.05 ± 1.26	NS
	physical	3.58 [2.65–4.83]	3.79 [2.69–4.80]	3.69 [2.69–4.76]	NS
	emotional	4.18 ± 1.36	4.27 ± 1.55	4.17 ± 1.35	NS
	social	3.92 [3.00–5.23]	4.08 [2.75–5.38]	3.92 [3.08–5.08]	NS
	Global Score	4.05 ± 1.29	3.98 ± 1.25	3.88 ± 1.30	NS
	physical	3.58 [2.65–4.83]	3.15 [2.62–4.69]	3.69 [2.52–4.65]	NS
	emotional	4.18 ± 1.36	4.10 ± 1.38	4.04 ± 1.44	NS
	social	4.13 ± 1.43	4.07 ± 1.41	3.96 ± 1.41	NS
Calcium 24-h urinary excretion: (mmol/24 h)	Global Score	4.05 ± 1.29	3.79 ± 1.42	3.98 ± 1.24	NS
	physical	3.58 [2.65–4.83]	3.15 [2.63–4.00]	3.69 [2.58–4.83]	NS
	emotional	4.18 ± 1.36	3.92 ± 1.69	4.14 ± 1.31	NS
	social	4.13 ± 1.43	3.74 ± 1.50	4.09 ± 1.39	NS
Phosphates (urine): (mg/dL)	Global Score	4.05 ± 1.29	4.06 ± 1.20	3.70 ± 1.40	NS
	physical	3.58 [2.65–4.83]	3.64 [2.80–4.69]	2.83 [2.40–4.72]	NS
	emotional	4.18 ± 1.36	4.23 ± 1.32	3.83 ± 1.53	NS
	social	4.13 ± 1.43	4.13 ± 1.34	3.79 ± 1.53	NS
Phosphates 24-h urinary excretion: (g/24 h)	Global Score	4.05 ± 1.29	4.34 ± 1.26	3.76 ± 1.30	0.031
	physical	3.58 [2.65–4.83]	4.08 [2.92–5.00]	3.12 [2.58–4.42]	0.027
	emotional	4.18 ± 1.36	4.41 ± 1.26	3.93 ± 1.48	0.093
	social	3.92 [3.00–5.23]	4.46 [3.31–5.54]	3.50 [2.79–4.73]	0.019
PTH (serum): (pg/mL)	Global Score	4.05 ± 1.29	4.15 ± 1.21	4.06 ± 1.32	NS
	physical	3.58 [2.65–4.83]	3.15 [2.73–4.96]	3.69 [2.69–4.83]	NS
	emotional	4.18 ± 1.36	4.36 ± 0.96	4.17 ± 1.41	NS
	social	3.92 [3.00–5.23]	3.31 [3.12–5.50]	3.92 [3.00–5.31]	NS

*Ca, calcium; PTH, parathormone*

### Factors predicting HRQoL in patients with hemodynamically significant MR

To determine the influence of patients’ age and gender on HRQoL in the studied population, we used dual linear regression. Based on the obtained results, we identified a significant influence of gender on MacNew emotional domain. Higher scores were observed in men (r = 0.730; 95% CI, 0.160–1.300; *p* = 0.014). We did not find significant relationships between age, gender, and other domains.

**Fig. 1:**
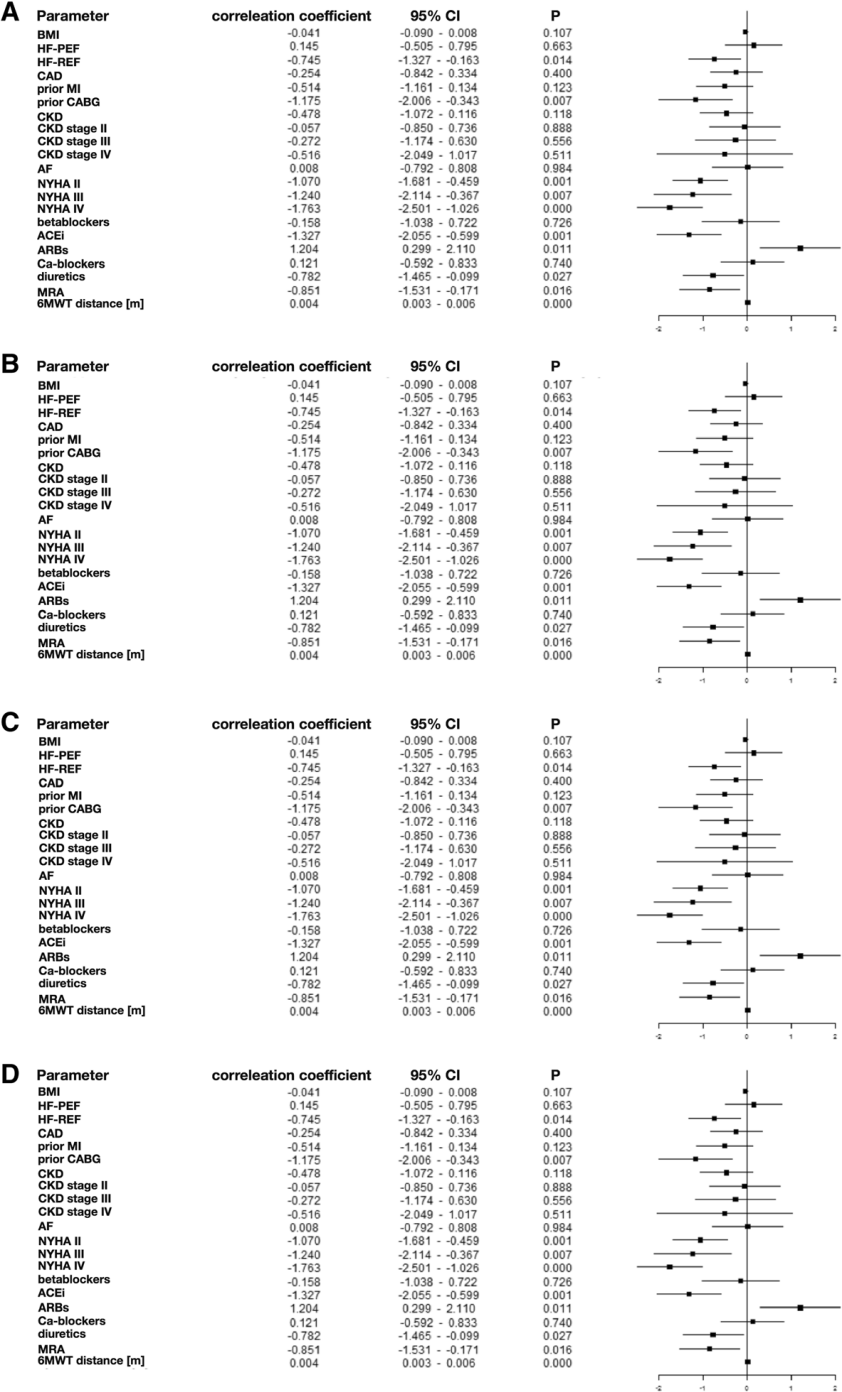
The influence of clinical parameters on HRQoL. **a** Global score, (**b**) physical domain, (**c**) emotional domain, (D) social domain. *BMI – body mass index, HF-PEF – heart failure with preserved ejection fraction; HF-REF – heart failure with reduced ejection fraction; CAD – coronary artery disease, MI – myocardial infarction; CABG – coronary artery bypass graft; CKD – chronic kidney disease; AF – atrial fibrillation; ACEi, angiotensin converting enzyme inhibitors; ARBs, angiotensin receptor blockers, MRA – mineralocorticoid receptor antagonist; 6MWT – 6-min walking test*

To ascertain the influence of clinical parameters on HRQoL we built a series of linear regression models considering age, gender, and chosen clinical parameter. We found that patients with higher NYHA HF class reported significantly poorer Global Scores (r = − 1.003, *p* = 0.001; r = − 1.018, *p* = 0.012; r = − 1.558, *p* < 0.001, for NYHA class II, III, and IV, respectively) and had poorer HRQoL in all other three domains (r = − 1.186, *p* < 0.001; r = − 1.092, *p* = 0.016; r = − 1.687, *p* < 0.001; r = − 0.833, *p* = 0.006; r = − 1.483, *p* < 0.001; r = − 1.070, p = 0.001; r = − 1.24, *p* = 0.007; r = − 1.763, *p* < 0.001 for NYHA class II, III, and IV, respectively, for physical, emotional, and social domains, respectively). Further analysis revealed that lower Global Scores and poorer HRQoL in all three domains resulted in shorter distance walked by patients during a 6 min walking test (r_1_ = 0.004, *p*_1_ < 0.001; r_2_ = 0.004, *p*_2_ < 0.001; r_3_ = 0.004, *p*_3_ < 0.001; r_4_ = 0.004, p_4_ < 0.001 for Global Scores, physical, emotional, and social domains, respectively) (Fig. 1).

To determine the influence of echocardiographic and biochemical parameters on HRQoL, we again built a series of linear regression models considering age, gender, and chosen echocardiographic or biochemical parameter. We concluded that patients with larger left ventricular systolic and diastolic volumes had lower Global Scores (r_1_ = − 0.006, 95% CI, 0.010 to − 0.002, p_1_ = 0.008; 95% CI, − 0.029 to − 0.003, r_2_ = − 0.011, p_2_ = 0.009 for LVES_vol._ and LVES_vol._ indexed for BSA, respectively, and r_1_ = − 0.004, 95% CI, − 0.008 to − 0.000, p_1_ = 0.045; r_2_ = − 0.008, 95% CI, − 0.015 to − 0.000, p_2_ = 0.049 for LVED_vol._ and LVED_vol._ indexed for BSA, respectively). We observed similar relationships for physical and social domains. Moreover, we showed that patients with higher NT-proBNP levels had poorer HRQoL in the physical and social domains (r_1_ = 0.000, 95% CI, − 0,000 to − 0,000, *p*_1_ = 0.036; r_2_ = 0.000, 95% CI, − 0.000 to − 0.000, *p*_2_ = 0.044).

## Discussion

To the best of our knowledge, our study is the first to assess the influence of selected Ca-P metabolism parameters on HRQoL in patients with significant MR. We also believe that it is the first study to identify factors determining HRQoL in this patient population.

The published data regarding HRQoL in patients with MR are derived from surgical cohorts of patients undergoing MV repair or replacement. In the analyzed papers, the authors indicated a significant influence of MR presence on HRQoL. They also found prominent HRQoL improvement in case of invasive treatment, despite the chosen surgical technique [5–12].

Due to the lack of published data to compare our results, we refer to two papers that discuss HRQoL in the context of Ca-P metabolism disturbances [21, 22]. One should keep in mind that in the cited papers, the authors presented different patient cohorts, and they used different HRQoL questionnaires. Nevertheless, in the paper by Siilin et al., the authors reported that premenopausal aged women with diagnosed Ca disturbances had poorer HRQoL in all assessed domains [21]. In the second article, the researchers studied the relationship between HRQoL and the frequency of hospitalizations and the markers of bone metabolism in patients with chronic kidney disease. They proved the presence of a correlation between poorer HRQoL and lower Ca levels as well as higher PTH levels [22]. In addition to presenting their own results, the authors directed attention to earlier studies that suggested a relationship between poorer HRQoL and Ca-P metabolism disturbances in patients who received dialysis [22]. The reported outcomes corresponded well with our results. In our cohort, poorer HRQoL, as assessed by Global Scores and scores in the physical and social domains, correlated significantly with higher PTH. Additionally, we showed that in the group of patients with elevated PTH levels, the mean Global Scores were significantly lower than those in the group with PTH levels within the normal range. Also, the median scores in the physical and social domains were significantly lower in the subgroup with elevated PTH levels. Furthermore, in the study population, the lower the vitamin D level, the poorer HRQoL was reported by patients in the physical domain.

As mentioned previously, there are no publications in the literature that would identify, in the context of Ca-P metabolism disturbances, factors determining HRQoL in patients with significant MR. To gain a better understanding of the significance of HRQoL impairment in our cohort, we related this impairment to patients suffering from HF because all the patients in our study had this condition diagnosed. In patients with HF, the main clinical symptoms that reduce activities of daily living and contribute to exercise intolerance are dyspnea, tiredness, and fatigue. HRQoL in patients with HF may be worsened by psychological problems, physical symptoms, social limitations, and adverse treatment effects. The increasing severity of HF may lead patients to realize their own mortality, which can result in depression, sleep disturbances, and anxiety [23, 24]. The results reported by Dunderdale et al. and Berry et al. are consistent with our outcomes. We found that patients with higher NYHA HF class reported significantly poorer Global Scores and had poorer HRQoL in all other three domains. Moreover, we showed that patients with higher NT-proBNP levels had poorer HRQoL in the physical and social domains. We found corresponding results regarding echocardiography. Patients with larger left ventricular systolic and diastolic volumes had lower Global Scores. We observed similar relationships for the physical and social domains. Further analysis revealed that lower Global Scores and poorer HRQoL in all three domains resulted in shorter distance walked by patients during a 6-min walking test.

### Study limitations

There are several limitations in the present study. First, the cohort size was rather small, which resulted in only 38 patients with severe MR. Second, we obtained Ca-P metabolism parameters only once during the study period. We were unable to determine several parameters, e.g., different vitamin D metabolites. Having those results would allow for a more detailed data analysis. As an additional limitation, we see the lack of long-term follow-up, including the follow-up after introducing Ca and vitamin D supplementation. Furthermore, the fact that all patients had HF might have been significant; however, more than a third of them were nearly asymptomatic. Another limitation is the lack of published data with which to compare our results, especially with respect to HRQoL.

## Conclusions

To the best of our knowledge, our report is the first to show that Ca-P abnormalities resulted in significantly worse HRQoL, especially in the physical domain, in a population of patients with hemodynamically significant MR.

## Abbreviations

HF: Heart failure
HRQoL: Health-related quality of life
IQR: Interquartile range
MR: Mitral regurgitation
MV: Mitral valve
PTH: Parathormone
